# The TUG-10 test detects exertional desaturation in patients with interstitial lung disease: correlation with 6-minute-walk-test, cardiopulmonary exercise test, and lung diffusion capacity

**DOI:** 10.1007/s00421-026-06207-8

**Published:** 2026-04-06

**Authors:** Marios Bismpos, Afroditi Boutou, Andreas Zafeiridis, Athanassios Zacharias, Stella Kritikou, Christina Rampiadou, Ioannis Gkalgkouranas, Leonidas Kastritseas, Ioannis Stanopoulos, Aikaterini Markopoulou, Georgia Pitsiou, Konstantina Dipla

**Affiliations:** 1 Laboratory of Exercise Physiology and Biochemistry, Department of Physical Education and Sport Sciences at Serres, Aristotle University of Thessaloniki, Serres, 62110 Greece; 2 Department of Respiratory Failure, G. Papanikolaou General Hospital, Aristotle University of Thessaloniki, Thessaloniki, Greece; 3 Department of Respiratory Medicine, G. Papanikolaou General Hospital, Thessaloniki, Greece; 4 School of Life and Health Sciences, University of Nicosia, Nicosia, Cyprus

**Keywords:** Interstitial lung diseases, Time-up-and-go-10, 6-minute-walk-test, Cardiopulmonary exercise test, Diffusing lung capacity, Exertional desaturation

## Abstract

**Background:**

The Time-Up-and-Go (TUG) test is clinical test, used to assess functional mobility in chronic diseases. A modified version, the TUG-10, involving 10 consecutive repetitions of the classic version, has been recently proposed to increase the physiological demand. However, TUG-10 ability to detect exertional desaturation in patients with interstitial lung disease (ILD) has not been investigated.

**Purpose:**

This study aimed to: (i) compare oxygen desaturation during the TUG-10 with 6-minute walk test (6MWT) and cardiopulmonary exercise test (CPET) in patients with ILD; (ii) examine correlations in exertional desaturation among these tests and with lung diffusing capacity (DL_CO_).

**Methods:**

Twenty patients with fibrotic-ILD (70.1 ± 7.3years) performed in random order the TUG-10, 6MWT, and CPET (on a cycle ergometer). Correlations between desaturation indices (SpO₂nadir and SpO₂magnitude, ΔSpO₂) across tests and with DL_CO_ were examined.

**Results:**

No significant differences were observed in desaturation among TUG-10, 6MWT, and CPET (ΔSpO_2_: -7.35 ± 3.8%, 7.0 ± 4.4%, and − 7.1 ± 3.7%, respectively; SpO2nadir: 89.2 ± 4.5%, 88.2 ± 5.2%, and 88.3 ± 4.6%). ΔSpO_2_ during TUG-10 correlated (*p* < 0.05) with ΔSpO_2_ in 6MWT (*r* = 0.62) and CPET (*r* = 0.61). SpO₂nadir during TUG-10 correlated (*p* < 0.001) with SpO₂nadir in 6MWT (*r* = 0.73) and CPET (*r* = 0.65). TUG-10 duration (84.4 ± 20.1s) correlated with 6MWT distance (*r*=-0.83; *p* < 0.001). SpO₂nadir during TUG-10 correlated with DL_CO_ (*r*=-0.68; *p* < 0.001).

**Conclusion:**

The TUG-10 is a brief (< 1.5 min) test that can induce exertional desaturation in patients with ILD. TUG-10 desaturation indices strongly correlate with those in 6MWT and CPET, and with DL_CO_, making it a practical test for detecting exertional desaturation, especially in primary care or private practice with limited equipment and space.

## Introduction

Interstitial Lung Diseases (ILD) constitute a heterogeneous group of pulmonary disorders, characterized by inflammation and/or fibrosis of the lung parenchyma (Smith and Jenkins, 2023; Wijsenbeek et al. 2022; Zeng and Jiang 2023). Pathophysiological characteristics of ILD are gas exchange impairments, ventilation-perfusion mismatch, and significantly reduced diffusion capacity, all of which limit the patients’ exercise capacity (Bonini and Fiorenzano 2017; Dipla et al. 2023; Molgat-Seon et al. 2019). These mechanisms exacerbate symptoms experienced by patients, such as dyspnea, early-onset fatigue, and exercise-induced desaturation, progressively resulting in low exercise tolerance (Dowman and Holland 2024; Marillier et al. 2023; Wickerson et al. 2020).

The comprehensive assessment of resting pulmonary function (including spirometry, static lung volumes, and diffusing capacity) is essential for the diagnosis and disease monitoring in ILD (Bonini and Fiorenzano 2017; Gille and Laveneziana 2021). However, these tests do not evaluate patients’ exercise capacity or responses to daily physical activities (Gille and Laveneziana 2021; Harari et al. 2022; Herdy et al. 2016). For this reason, exercise tests serve as valuable tools for assessing functional capacity and mobility, monitoring disease progression, and identifying impairments in gas exchange, including significant exertional oxygen-hemoglobin desaturation (< 88%) (Briand et al. 2018; Tomlinson et al. 2022). Moreover, significant exertional desaturation assesses the severity of gas exchange impairments and is also recognized as a prognostic marker of mortality in patients with ILD (Barratt et al. 2020; Oğuz et al. 2024; Otake et al. 2023).

The Cardiopulmonary Exercise Test (CPET) is considered the gold standard exercise test for assessing maximal aerobic capacity and exercise intolerance (Palange et al. 2007). This maximal or symptom-limited test provides a comprehensive physiological assessment of the respiratory, cardiovascular, and muscular system responses during exercise in a controlled laboratory environment and enables the identification of the primary physiological factors limiting exercise performance (ATS 2003; Barratt et al. 2020). Peak oxygen uptake (VO_2peak_) in CPET is a strong prognostic marker of mortality in patients with ILD (Bonini and Fiorenzano 2017; ATS 2003; Barratt et al. 2020; Oğuz et al. 2024; Stubbe et al. 2022; Tomlinson et al. 2022). However, the CPET requires specialized equipment, qualified personnel and is time-consuming (ATS 2003), therefore, it is not always available in clinical practice.

The 6-Minute Walk Test (6MWT) is a widely used, self-paced aerobic field test that assesses submaximal exercise capacity and functional performance (Albarrati et al. 2016; ATS 2002; Singh et al. 2014). Although the test does not distinguish between contributing elements to exercise limitation, the distance covered during the 6MWT reflects the overall functional status of patients with ILD, as it is influenced by lung disease severity as well as frailty, deconditioning, and musculoskeletal impairment (Holland et al. 2014) and has shown prognostic value (Caminati et al. 2009; Kawut et al. 2005; Kim et al. 2024; Stubbe et al. 2022; Triantafillidou et al. 2013). Specifically, lowest oxygen saturation (SpO₂nadir) during 6MWT correlates with VO_2peak_ in IPF patients and a value < 88% is a significant predictive factor of mortality, while the magnitude of desaturation (ΔSpO_2_) is associated with the longitudinal changes in lung function and in diffusion capacity (FVC and DL_CO_) in ILD (Kim et al. 2024; Lama et al. 2003). The 6MWT is technically easier to perform than CPET, however, it requires a 30-meter corridor, which is not always available in primary care facilities or private practices (ATS 2002; Briand et al. 2018; Nakazawa et al. 2023).

Summarizing, both 6MWT distance and VO₂peak from CPET are strong predictors of mortality and provide important functional information for patients with ILD (Barratt et al. 2020; Caminati et al. 2009; Oğuz et al. 2024; Stubbe et al. 2022; Tomlinson et al. 2022), however, they may be challenging to implement in daily clinical practice. For this reason, functional performance tests, such as the Time-Up-and-Go (TUG) or the Sit-to Stand tests (Briand et al. 2018; Liwsrisakun et al. 2020; Mesquita et al. 2016; Zamboti et al. 2021) have been proposed. The TUG is a simple, agility test that has been used as a prognostic marker of mortality in older adults, as well as in other populations with chronic disease (Ascencio et al. 2022; Hendriks et al. 2022). The TUG test has been used to assess differences in functional performance between patients with chronic obstructive pulmonary disease (COPD) and controls (Albarrati et al. 2016) and to detect balance impairments (Liwsrisakun et al. 2020). Furthermore, the duration of the classic TUG test version has been negatively correlated with the 6MWT distance in patients with chronic heart failure (Hwang et al. 2016) and COPD (Albarrati et al. 2016). However, a single repetition of the TUG test is typically of insufficient duration and physiologic demand to reliably provoke exercise-induced desaturation.

The 10-repetition TUG test (TUG-10) has been recently proposed as a more demanding variation of the test (Hergott et al. 2022), as performing 10 continuous repetitions adds an endurance component and increases the overall physiological demand. It has not been examined to date whether the TUG-10, given its relatively brief duration (1–1.5 min), can detect desaturation during exertional effort in patients with ILD. Additionally, it has not been examined previously whether the desaturation exhibited during this test correlates with lung diffusing capacity (DL_CO_) in patients with ILD.

Therefore, the aims of this study were to (i) compare the desaturation (magnitude of decline, ΔSpO_2_ and lowest values, SpO_2_nadir) in TUG-10 with that in 6MWT and CPET, and examine correlations in desaturation among the three tests, and the level of agreement in these measurements; (ii) investigate correlations between functional performance outcomes (TUG-10 duration and 6MWT distance), with CPET-derived physiological and performance outcomes (VO₂peak and Workpeak) and (iii) examine the correlation of TUG-10 desaturation with DL_CO_ (% predicted values), in patients with ILD. We hypothesized that the TUG-10 would induce exertional desaturation in patients with ILD, and that this desaturation would correlate with the respective values in 6MWT and CPET, and with DL_CO_ values.

## Methods

### Participants

Twenty patients (70.1 ± 7.3 years, 13 males, 7 females) with fibrotic-ILD phenotypes participated. The participants were patients attending the ILD outpatient clinic/pulmonary rehabilitation program or the Respiratory Failure Clinic from 5/2024 to 5/2025. The study protocol was approved by the Institution’s Human Research Ethics Committee and performed according to the Declaration of Helsinki (2013 Amendment). Prior to enrollment, participants were informed of the testing procedures and provided written consent. Inclusion criteria: (i) patients with fibrotic-ILD, without resting hypoxemia (SpO_2_ >93%), on anti-fibrotic medication (nintedanib or pirfenidone) at least for three months prior to the study, (ii) no change in medication and/or hospitalization due to respiratory failure or respiratory infection during the past three months. Exclusion criteria: (i) severe exacerbation in the last three months, (ii) presence of known resting pulmonary hypertension (as assessed by right heart catheterization, with Mean Pulmonary Arterial Pressure ≥ 20 mmHg), (iii) uncontrolled diabetes mellitus, uncontrolled hypertension, or severe heart failure, (iv) presence of absolute contraindications for CPET. On testing days, participants were instructed to report to the laboratory after a 3-hour fast, to refrain from caffeine and smoking for at least 8 h prior to the test, and alcohol and any form of intense physical activity for at least 24 h prior to the assessment.

### Study design

This is a prospective observational study. After enrolment, participants underwent a standardized clinical examination and respiratory function assessment, including spirometry, static lung volumes, and gas exchange assessment. Participants subsequently completed the 6MWT, the maximal CPET on a cycle ergometer, and the TUG-10 test. The order of the tests was randomized using an online randomization program. The functional tests were separated by 5 days. The SpO_2_ was recorded throughout the tests. Tests were performed under the supervision of a pulmonologist and an exercise physiologist. Correlations between desaturation indices (SpO₂nadir and ΔSpO₂) across the three tests and with DL_CO_ were examined. In addition, correlations between performance outcomes (TUG-10 duration and 6MWT distance) and CPET-derived physiological (VO_2peak_) and performance (peak work rate and duration) variables were examined.

### Experimental procedure-measurements

Upon arrival at the laboratory, participants were familiarized with the experimental procedures. Spirometry was performed prior to the exercise tests. Next, resting values of heart rate and blood pressure were recorded; SpO₂ was continuously monitored using a finger pulse oximeter (Microlife OXY 300, Microlife Corporation, USA). Participants then performed one of the three exercise tests (TUG-10, CPET, or 6MWT) in a random order.

*Cardiopulmonary Exercise Testing (CPET)* Participants underwent a maximal exercise test on a cycle ergometer. Respiratory gas exchange was recorded by a metabolic cart (Medgraphics, Ultima CPXTM, MGC Diagnostics). Calibration of the flow sensor and the gas analyzers was performed just prior to starting the CPET. The CPET protocol included a 3-min rest period for baseline measurements, followed by 2 min of unloaded cycling, the incremental exercise to exhaustion, and recovery. The 2-min unloaded phase was chosen in order to perform the incremental exercise phase before inducing a significant amount of fatigue, especially in the IPF patients with low DL_CO_ (Radtke et al. 2019). The incremental exercise was performed using a progressive RAMP protocol, with linear increase in work rate of 10 W/min. The selected work rate increment allowed the duration of the incremental exercise phase in ILD patients to be approximately 10 min (ranging between 8 and 12 min) (Radtke et al. 2019). Patients were instructed to maintain a steady cadence (50–60 rpm) and encouraged to provide maximal effort. Blood pressure was recorded at 2-min intervals; a 12-lead electrocardiogram (CardioSoftTM, GE HealthCareTM, California, USA) and SpO_2_ were continuously monitored. The criteria for CPET termination were as described in the ATS/ACCP Statement on Cardiopulmonary Exercise Testing (Radtke et al. 2019; ATS 2003). The % predicted values for VO₂peak and peak work rate were obtained using age- and sex-adjusted equations as described in Wasserman and Hansen (2005).

*6-Minute Walk Test (6MWT)* The 6MWT was administered on a 30-meter-long, flat, straight corridor, as previously recommended (Holland et al. 2014, 2015). Briefly, participants were instructed to walk as far as they could in 6 min along the designated path. Encouragement was given every 60 s using standardized phrases. If the patient stopped walking during the test, the timer was not stopped. The patient was allowed to rest while sitting or standing, as preferred (Holland et al. 2014, 2015). While the patient was resting, standardized encouragement was provided every 30 s. The time that the patient stopped and the time that walking was recommenced was recorded. The total distance covered with rounding to the nearest meter and SpO_2_ values were recorded. Baseline and end test heart rate, as well as baseline and SpO_2_nadir were recorded; number of stops, duration, and symptoms during the test were also recorded.

*Time-Up-and-Go-10-repetitions (TUG-10) test* The TUG-10 was performed to assess functional mobility using a 3-meter marked path and a chair with armrests, positioned against the wall. As both males and females patients with different body heights participated, two standard chair heights were used, selected according to participant stature, to achieve approximately 90° knee flexion while seated. Participants started from a seated position. On the examiner’s signal, they were instructed to stand up, walk to a cone used as a marker point, turn around, return to the chair, and sit back down, repeating this process 10 times without stopping (Hergott et al. 2022). The instruction was to perform the test “quickly and safely as possible”. For standardization, participants in our study were instructed to stand up and sit down without using their arms, unless absolutely necessary. This approach ensured comparable physical demand across all participants, which was important for assessing desaturation during the TUG-10. Participants performed one practice trial. Testing began on the instruction “1, 2, 3, go” and ended when the participant sat in the chair after the 10th repetition. At the end of each repetition, the number of repetitions completed was announced and a verbal feedback “you are doing well” was provided. Total completion time was recorded using a digital stopwatch, while SpO_2_ and heart rate were continuously monitored using pulse oximetry.

### Statistical analysis

For statistical analysis, SPSS software version 29.0 (IBM Corp., Armonk, NY, USA) was used. Data are presented as mean ± standard deviation (mean ± SD) or median (interquartile range), depending on normality of distribution, assessed using the Kolmogorov-Smirnov test. Due to the lack of published data on TUG-10 in ILD, an a priori sample size or power calculation was not performed. Pearson’s or Spearman’s correlation was used, as appropriate, to examine associations among the variables of interest. The following variables were analyzed for potential correlations: (i) SpO_2nadir_ and ΔSpO_2_ (i.e. SpO_2rest_-SpO_2nadir_) across all three tests, as well as with DL_CO_ (% predicted), (ii) maximal oxygen uptake (VO_2peak_, ml/kg/min), peak work rate (Watt), test duration (min) from CPET, (iii) distance covered (m) during the 6MWT, and (iv) duration (s) of the TUG-10 test. Differences in oxygen desaturation indices among TUG-10, 6MWT, and CPET were examined using linear mixed-effects models with test condition as a fixed effect and participant as a random effect. This approach allows inclusion of all available observations and appropriately accounts for within-subject comparisons in the presence of missing data. Pairwise comparisons were adjusted for multiple comparisons using the Sidak correction. Agreement between the TUG-10, 6MWT, and CPET for SpO₂ nadir was evaluated using Bland-Altman analysis, with the mean difference representing systematic bias and the 95% limits of agreement calculated as ± 1.96 standard deviations; proportional bias was assessed by correlating the mean and difference of each test pair. Statistical significance level for all analyses was set at α = 0.05.

## Results

### Participants’ characteristics and pulmonary function tests

Twenty patients (58–80 years old) with fibrotic-ILD, on anti-fibrotic medication (Nintedanib *n* = 17, Pirfenidone *n* = 3) participated in this study. The anthropometric characteristics, baseline hemodynamics, co-morbidities, and pulmonary function assessment data are presented in Table 1. Participants had impaired diffusing capacity (*DL*_CO_) with values ranging from 30 to 79% of predicted.

**Table 1:** Participants’ characteristics

Anthropometrics	Mean ± SD
Age (years)	70.1 ± 7.3
Height (m)	1.65 ± 0.10
Weight (kg)	76.8 ± 10.7
Body mass index (kg/m2)	27.3 ± 4.3
Males/females	13/7
*Baseline (resting) hemodynamics*	Mean ± SD
Systolic Blood Pressure (mmHg)	121.9 ± 12.8
Diastolic Blood pressure (mmHg)	78.0 ± 8.2
Heart rate (bpm)	79.4 ± 13.1
*Resting pulmonary function testing*	Mean ± SD
FVC (L)	2.99 ± 1.03
FVC (% predicted)	90.42 ± 18.99
FEV1	2.36 ± 0.66
FEV1 (%predicted)	90.89 ± 17.56
FEV1/FVC	78.85 ± 7.5
FEV1/%FVC	102.56 ± 9.80
VC (L)	3.00 ± 1.03
VC (% predicted)	90.40 ± 19.97
DL_CO_ (mL/min/mmHg)	10.46 ± 3.82
DL_CO_ (% predicted)	49.10 ± 14.04
*Diagnosis*	(n=)
Idiopathic Pulmonary Fibrosis	16
Idiopathic fibrotic non-specific interstitial pneumonia	2
Connective tissue related ILD	2
*Co-Morbidities*	(n=)
Cardiovascular disease	7
Renal disease	2
*Smoking status*	(n=)
Current/Previous/Never	1/15/6

*DL*_*CO*_ lung diffusing capacity for carbon monoxide, *VC* vital capacity, *FVC* forced vital capacity, *FEV1* forced expiratory volume in 1 s

### Comparison of oxygen desaturation across exercise tests

**Fig. 1:**
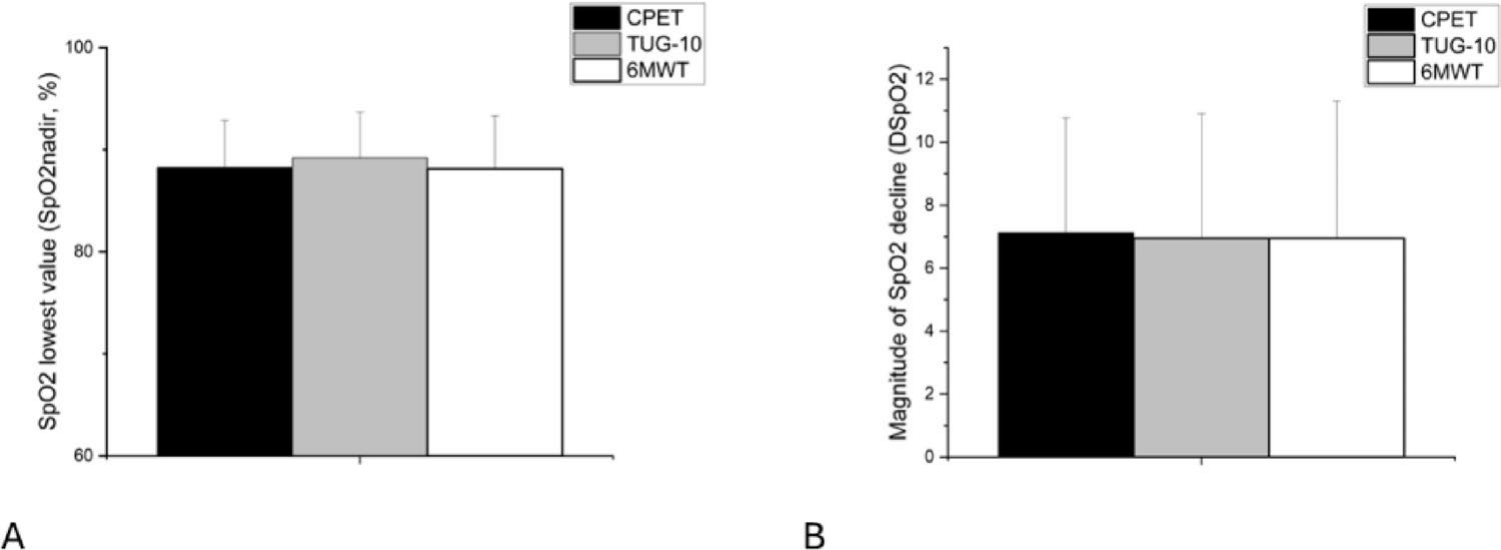
Comparisons of desaturation indices across the cardiopulmonary exercise test (CPET), Time-Up-and-Go-10-repetitions (TUG-10) test, and 6-Minute Walk Test (6MWT). **A** Lowest value in hemoglobin oxygen saturation (SpO_2_nadir) and **B** Magnitude of decline in SpO_2_ (DSpO_2_)

Table 2 presents the performance characteristics and the oxygen-hemoglobin desaturation observed during each test. Of the 20 participants, four participants did not complete the maximal CPET (two due to musculoskeletal or hemodynamic limitations and two due to acute illness). Thus, comparisons of desaturation indices (i.e. SpO_2nadir_ and ΔSpO_2_) across the TUG-10, 6MWT, and CPET were analyzed using a linear mixed-effects model to account for the missing observations. No statistically significant differences among CPET, 6MWT, and TUG-10 test were detected in SpO_2nadir_ (*p* = 0.31) and ΔSpO_2_ (*p* = 0.946) (Fig. 1A and B, respectively).

**Table 2:** Results of the Time-Up-and-Go-10-repetitions (TUG-10) test, Cardiopulmonary Exercise Testing (CPET), and 6-Minute Walk Test (6MWT)

	TUG-10 Mean ± SD	CPET Mean ± SD	6MWT Mean ± SD
Duration (min)	1.43 ± 0.3	9.2 ± 2.8	6.0 ± 0
SpO_2_rest (%)	96.2 ± 1.6	95.4 ± 2.4	95.1 ± 2.0
SpO_2_nadir (%)	89.2 ± 4.5	88.3 ± 4.6	88.2 ± 5.2
ΔSpO_2_ (%)	6.7 ± 4.0	7.1 ± 3.7	7.0 ± 4.4
Heart rate rest (bpm)	74.0 ± 11.8	78.4 ± 13.0	80.3 ± 11.3
Heart rate peak (bpm)	110.7 ± 17.9	126.9 ± 19.4**	107.3 ± 16.2
VO_2_peak, ml/kg/min (% predicted)	-	18.2 ± 4.2 (80.4 ± 19.3)	-
Peak work rate, W (% predicted)	-	92.4 ± 28.0 (80.2 ± 22.8)	-
Distance (m)	-	-	441.5 ± 91.1

* SpO*_2_*nadir* lowest oxygen hemoglobin desaturation during the test, *ΔSpO*_2_ Magnitude of oxygen hemoglobin desaturation from pulse oximetry during the test

**p < 0.001 vs. TUG-10 and 6MWT

### Correlation of exercise-desaturation across the exercise tests and levels of agreement

**Fig. 2:**
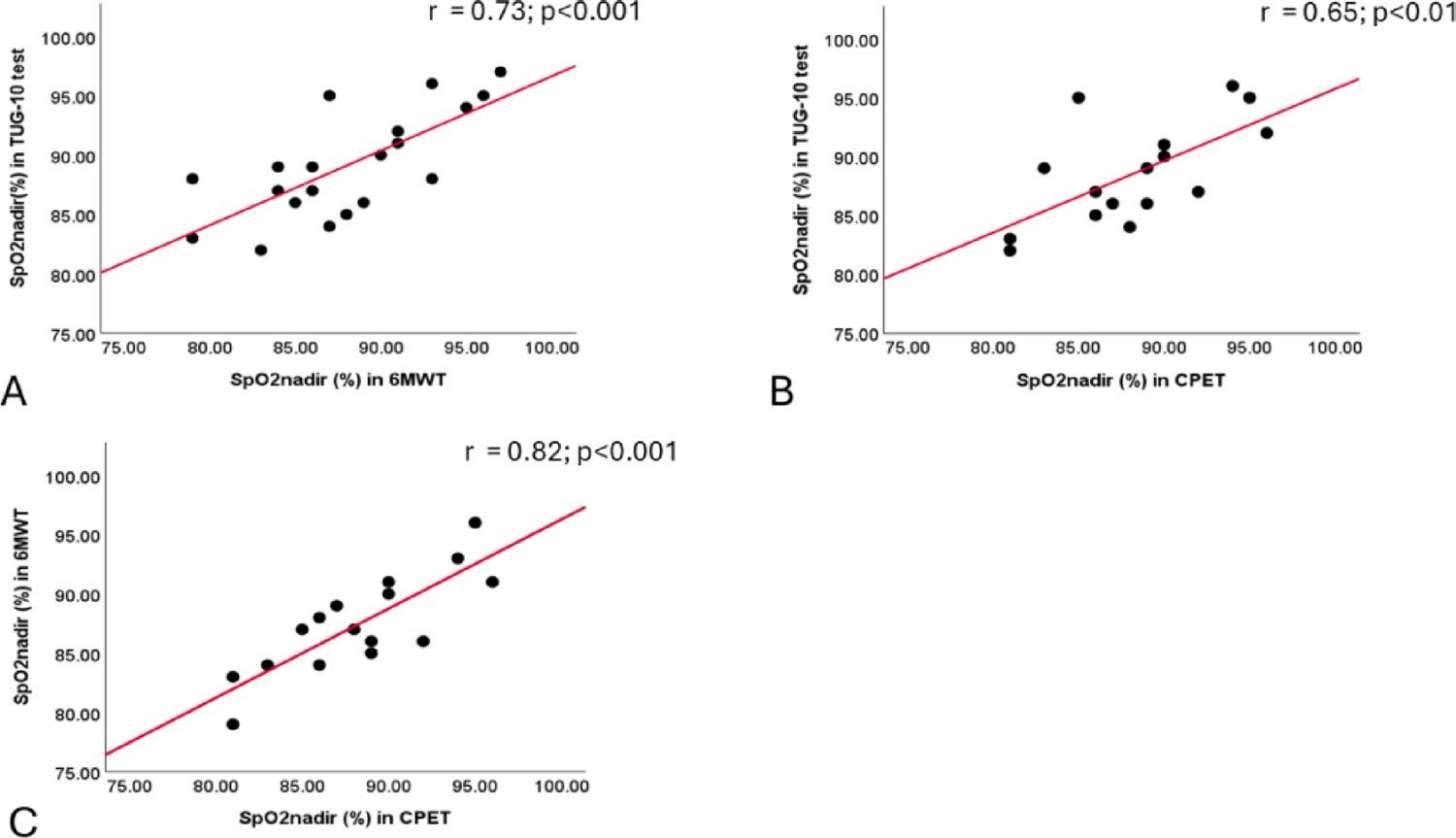
Correlations in lowest value in hemoglobin oxygen saturation (SpO_2_nadir) between **A** Time-Up-and-Go-10-repetitions (TUG-10) test and 6-Minute Walk Test (6MWT), **B** Time-Up-and-Go-10-repetitions (TUG-10) test and cardiopulmonary exercise test (CPET), and **C** 6-Minute Walk Test (6MWT) and cardiopulmonary exercise test (CPET),

The SpO_2_nadir values recorded during the TUG-10 test exhibited a strong positive correlation with those measured during 6MWT (*r* = 0.725, *p* < 0.001; Fig. 2A) and during CPET (*r* = 0.645, *p* < 0.01; Fig. 2B). A strong positive correlation was observed between SpO_2_nadir values in the 6MWT and CPET (*r* = 0.823, *p* = 0.001; Fig. 2C).

The ΔSpO_2_ during the TUG-10 test exhibited a strong correlation with the respective one in CPET (*r* = 0.605, *p* < 0.05) and in 6MWT (*r* = 0.620, *p* < 0.05), as presented in Table 3.

**Table 3:** Pearson (r) correlations between performance and desaturation indices in the Time Up-and-Go-10-repetition (TUG-10) test and corresponding indices from cardiopulmonary exercise testing (CPET) and the 6-Minute Walk Test (6MWT)

	CPET					6MWD		
	VO₂peak (ml/kg/min)	Duration (min)	Peak work rate, (W)	ΔSpO_2_ (%)	SpO_2_nadir (%)	Distance (m)	ΔSpO_2_ (%)	SpO_2_nadir (%)
TUG-10 duration (s)	−0.394	−0.255	−0.383	−0.112	0.001	−0.830**	−0.327	0.194
TUG-10 ΔSpO_2_ (%)	−0.084	−0.289	−0.139	0.605*	−0.577*	0.062	0.620**	−0.633**
TUG-10 SpO_2_nadir (%)	−0.126	0.181	0.120	−0.577*	0.645**	0.128	−0.642**	0.725**

*SpO*_2_*nadir* lowest saturation during the test, *ΔSpO*_2_ magnitude of desaturation during the test

*p < 0.05, **p < 0.01

**Fig. 3:**
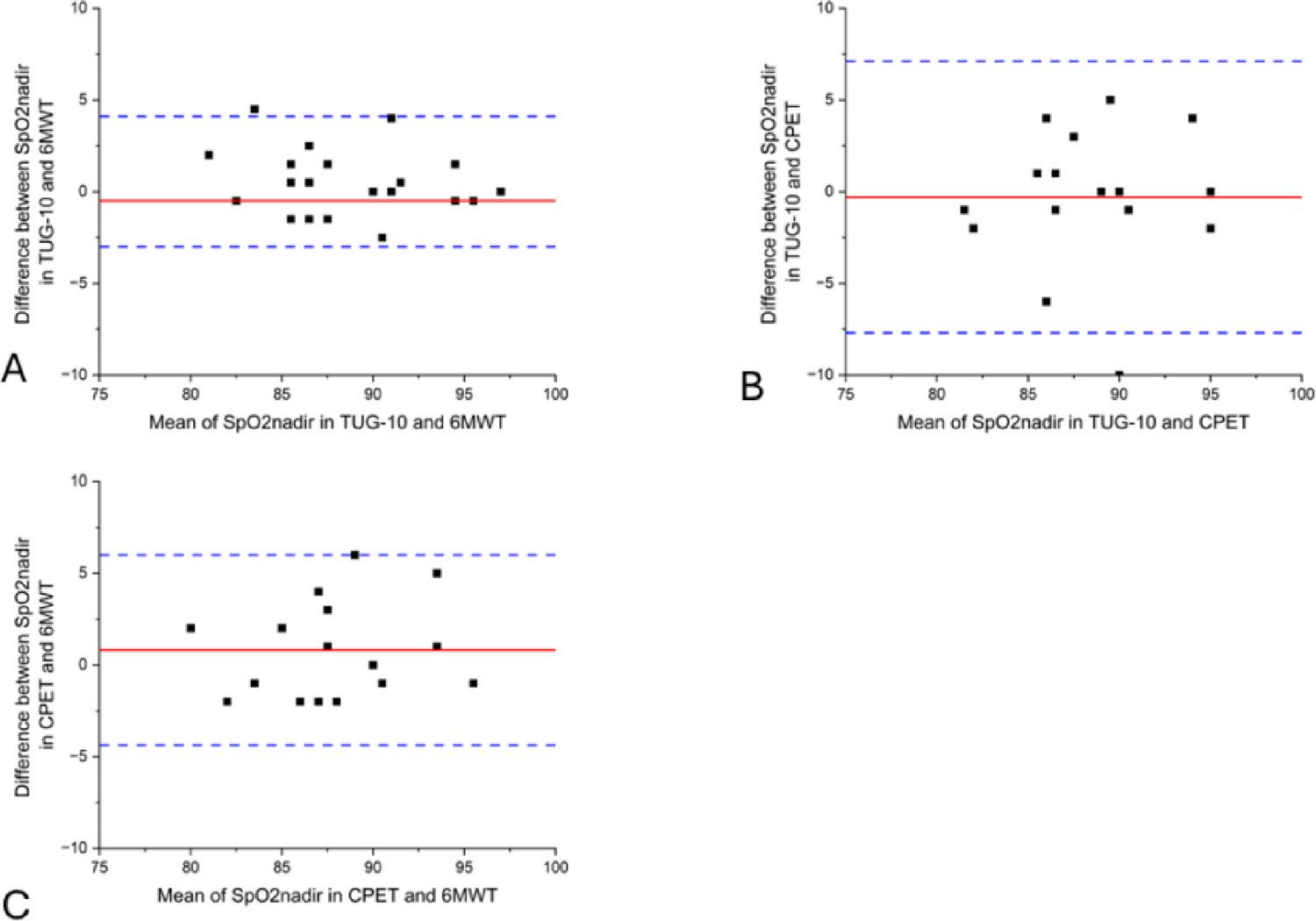
Bland–Altman plots showing agreement in oxygen hemoglobin saturation lowest value (SpO₂nadir) between **A** Time-Up-and-Go-10-repetitions (TUG-10) and 6-min Walk Test (6MWT), **B** TUG-10 and cardiopulmonary exercise test (CPET) and **C** CPET and 6MWT. Solid red lines represent mean differences (mean bias), and dashed blue lines indicate 95% limits of agreement (± 1.96 SD)

Agreement between desaturation indices measured during the TUG-10, 6MWT, and CPET was assessed using Bland-Altman analysis; the results are shown in Fig. 3. For SpO₂nadir, Bland-Altman analysis showed minimal bias between TUG-10 and 6MWT (mean bias = 0.5%), with 95% limits of agreement between − 3.0 and 4.1%; minimal bias was also detected between TUG-10 and CPET (mean bias = −0.31, 95% limits of agreement − 7.70 to 7.1%). The CPET showed a small positive bias relative to 6MWT (mean bias = 0.8%, 95% limits of agreement − 4.4 to 6.0%).

For ΔSpO₂, Bland-Altman analysis showed minimal bias between TUG-10 and 6MWT (mean bias = + 0.4%, with 95% limits of agreement from − 5.7% to + 6.5%) and minimal bias between TUG-10 and CPET (mean bias = −0.6%, with 95% limits of agreement from − 5.0% to + 6.2%). A small bias was observed between CPET and 6MWT for ΔSpO₂ (mean bias = −1.3% and 95% limits of agreement from − 5.7% to + 6.5%).

### Correlation of functional performance indices and physiologic measures

Correlation of performance indices and physiological indices between the exercise tests are presented in Table 3. The duration of the TUG-10 test exhibited a strong negative correlation (*p* < 0.001) with the total distance covered during the 6MWT. In contrast, the duration of the TUG-10 test did not show a statistically significant correlation with CPET duration, and peak work rate. A significant correlation (*p* < 0.05) was observed between 6MWT distance and VO₂peak. No significant correlation was detected betweenTUG-10 duration and VO₂peak.

### Correlation of desaturation indices during the exercise tests with the lung diffusion capacity

The correlation between desaturation indices during TUG-10, CPET, and 6MWT with lung diffusing capacity are presented in Table 4. The SpO_2_nadir during the TUG-10 test showed a strong correlation with DL_**CO**_ % predicted values (*r* = 0.683, *p* < 0.001). In addition, ΔSpO_2_ during the TUG-10 test showed a negative correlation with the DL*CO* % predicted values (*r*= −0.631, *p* < 0.01). Significant correlations were also detected between the desaturation indices in CPET and 6MWT with DL_CO_ % predicted values (Table 4). No significant correlations were detected between the desaturation indices in the three exercise tests and FVC, FEV1, or FEV1/FVC ratio.

**Table 4:** Pearson (r) correlations between desaturation indices in Time-Up-and-Go-10-repetition (TUG-10) test, cardiopulmonary exercise testing (CPET), and 6-Minute Walk Test (6MWT) and lung diffusing capacity

Exercise test	DL_CO_ (% predicted)
*TUG-10*	
ΔSpO_2_ (%)	−0.631*
SpO_2_nadir (%)	0.683**
*CPET*	
ΔSpO_2_ (%)	−0.705**
SpO_2_nadir (%)	0.706**
*6MWT*	
ΔSpO_2_ (%)	−0.726**
SpO_2_nadir (%)	0.747**

* DLCO* diffusing capacity of the lung for carbon monoxide, *SpO*_2_*nadir* lowest saturation during the test, *ΔSpO*_2_ magnitude of desaturation during the test

*p < 0.01, **p < 0.001

## Discussion

To our knowledge, this is the first study to compare exercise-induced desaturation across the TUG-10, 6MWT, and CPET and to examine the correlations between these tests in terms of desaturation, performance measures, and *DL*_CO_ % predicted in patients with ILD. Our findings were (i) the magnitude of oxygen-hemoglobin desaturation during the TUG-10 test was comparable to that observed in the 6MWT and CPET with strong correlations and good agreement between desaturation parameters (SpO_2_nadir and ΔSpO_2_) across the three tests and minimal systematic bias, supporting their comparability for assessing exercise-induced desaturation, (ii) the duration of the TUG-10 test exhibited a strong negative correlation with the total 6MWT distance covered, however, it was not significantly correlated with the CPET-derived measures of aerobic capacity (VO₂peak) or performance (Workpeak) (iii) strong negative correlations were observed between TUG-10 desaturation indices and %predicted DL_CO_.

### Oxygen-hemoglobin desaturation across tests

In this study, desaturation observed during the TUG-10 was comparable to and strongly correlated with that measured during the 6MWT and during CPET, suggesting that this brief functional test can elicit exertional desaturation in patients with ILD. Both desaturation indices (SpO₂nadir and ΔSpO₂) obtained during the TUG-10 and 6MWT were strongly correlated with corresponding CPET values, supporting previous findings that SpO₂nadir and ΔSpO₂ during submaximal field tests reflect gas-exchange impairment, similarly to maximal exercise testing (Holland et al. 2014; Tremblay Labrecque et al. 2020). The Bland-Altman analysis confirmed the above findings showing acceptable agreement in desaturation indices between tests, with small mean biases, and no clinically meaningful systematic differences. Overall, SpO₂nadir exhibited slightly narrower limits of agreement than ΔSpO₂, indicating marginally greater consistency of this index, especially when comparing the TUG-10 with the 6MWT.

*Comparison of TUG-10 desaturation with the 6MWT* Both the 6MWT and TUG-10 are submaximal, self-paced field tests of functional performance. Despite TUG-10 brief duration, its physiological demand appears comparable to that of the 6MWT. Although neither field test elicited the maximal heart rates achieved during CPET, the TUG-10 produced peak heart rates and desaturation (magnitude and nadir) similar to those observed during the 6MWT. Previous studies have evaluated exercise-induced desaturation during other functional tests, such as 1-min sit-to-stand test (1-min-STS), in comparison with the 6MWT. Specifically, Briand et al. (2018) reported that in patients with ILD, the 1-min-STS elicited less desaturation than that observed during the 6MWT. In contrast, our findings suggest that the combined sit-to-stand and walking components of the TUG-10 impose a greater physiological demand than the 1-min-STS, resulting in similar desaturation to the 6MWT. In support of this finding, previous evidence suggests that the magnitude of desaturation during field tests depends not only on exercise duration but also on the nature and intensity of the task (Holland et al. 2014). Holland et al. (2014) reported that although the 6MWT elicited lower VO_2_ values compared with the CPET, the desaturation during the 6MWT (i.e. during walking) exceeded that observed during maximal cycling exercise in patients with ILD. Hofman et al. (2024) also reported that short, fast daily activities induce desaturation of similar or even greater magnitude than the 6MWT (Hoffman et al. 2024).

In more detail, the TUG-10 may be physically harder from its onset. It incorporates multiple demanding functional components, including repeated sit-to-stand maneuvers, frequent turning, and brisk walking, which require rapid force generation, balance, and coordination, potentially increasing both the demand and oxygen utilization from the onset of the test. In addition, sit-to-stand transitions require lower-limb power and upper body muscle stabilization, possibly increasing sensitivity to detect muscle dysfunction. Patients with ILD often present reduced muscle strength and endurance, particularly in the lower limbs (Garcia et al. 2024) which may limit oxygen utilization during exercise contributing to early fatigue (Dipla et al. 2021; Marillier et al. 2023). The decline in endurance potentially increases the anaerobic contribution of the TUG-10 and possibly enhances the rate of desaturation. Collectively, these factors may explain why the TUG-10, despite being brief, can elicit desaturation similar to the longer 6MWT field test.

*Comparison of TUG-10 desaturation with CPET* The CPET is an incremental test designed to assess maximal aerobic capacity, whereas, as mentioned above, the TUG-10 is a brief, self-paced functional performance test that does not typically reach maximal heart rate. Nevertheless, both tests induced comparable desaturation. A possible explanation is that during CPET the cardiopulmonary load progressively increases and peak physiological responses are elicited at the end of the test, whereas, the TUG-10 imposes rapid movements from the onset, requiring acceleration, balance, and muscular effort; this abrupt demand may accelerate the rate of O_2_ utilization and thus, induce faster desaturation in patients with limited gas-exchange reserve. Thus, despite its short duration, the rate of oxygen utilization may be greater during the TUG-10 compared to the initial stages of the CPET, which typically becomes more demanding in terms of oxygen utilization only during the final stages of the protocol. Furthermore, CPET does not involve a weight-bearing task, which may reduce its desaturation effect compared to the shorter, brisk-walking nature of the TUG-10 test.

### Correlation of desaturation indices with DL_CO_

To our knowledge, no studies have investigated the relationship between desaturation indices during the TUG-10 and diffusing capacity in patients with fibrotic-ILD. This study shows a strong inverse correlation between desaturation during the TUG-10 and percent predicted DL_CO_, suggesting that greater desaturation during the test reflects more severe gas exchange impairment. These findings agree with previous studies reporting significant associations between exercise-induced desaturation during the 6MWT or the 1-min STS and DL_CO_ in patients with ILD (Alfieri et al. 2020; Nishiyama et al. 2016; Triantafillidou et al. 2013; Wallaert et al. 2012). In fibrotic-ILD, thickening of the alveolar-capillary membrane and fibrosis of the lung parenchyma lead to ventilation-perfusion mismatch, which becomes more pronounced when the metabolic demands increase (Bonini and Fiorenzano 2017). Even during brief exertion, impaired diffusion and ventilation-perfusion mismatch do not allow sufficient arterial oxygenation, resulting in a drop of SpO_2_ that reflects diffusion limitations (Bonini and Fiorenzano 2017). In previous studies, desaturation during walking or during functional tests was strongly correlated with lower DL_CO_ values that were not captured in resting spirometry values such as FVC (Oishi et al. 2022). The present results extend these findings by showing that the TUG-10 test can uncover clinically meaningful desaturation that corresponds to underlying impairment in respiratory gas exchange and can be used in settings where more extensive testing is not feasible. Future studies are needed to validate these findings in larger ILD populations and to explore the predictive value of TUG-10 desaturation in relation to clinical outcomes such as disease progression, quality of life, and mortality, as well as in response to rehabilitation.

### Correlation of performance parameters in TUG-10 and 6MWT with CPET-derived physiological and performance parameters

No significant correlations between functional performance in TUG-10 (i.e. TUG-10 duration) with CPET-derived parameters of aerobic capacity (VO_2peak_) and performance (peak work rate and CPET duration) were observed. This is possibly related to the distinct nature of the two tests: the CPET primarily evaluates maximal aerobic capacity under controlled, progressively increasing workloads; it was performed on a cycle ergometer, assessing cardiopulmonary responses during a non-weight-bearing activity and it requires lower-limb endurance. In contrast, the TUG-10 does not maximally stress the cardiopulmonary system, is a weight-bearing test involving rapid transitions between sitting, standing, and walking, which reflect functional performance during daily life activities. These findings suggest that the TUG-10 appears to reflect functional performance rather than maximal aerobic capacity, based on the variables examined in this study. In contrast, the duration of the TUG-10 test was strongly correlated with the total distance covered during the 6MWT. Both TUG-10 and 6MWT tests share a similar physical demand, including walking, lower limb muscle endurance, and balance. Unlike the classic TUG test, which involves a single repetition, the TUG-10 is a more demanding test that its performance is influenced by lower limb muscle endurance, gait speed, and dynamic balance, potentially enhancing its sensitivity in assessing functional performance and exertional desaturation. Previous studies have examined the classic version of the TUG test (one repetition) as a measure of physical function and a predictor of all-cause mortality in older adults(Ascencio et al. 2022) and in patients with COPD (Albarrati et al. 2016). These studies focused solely on test duration and reported that prolonged TUG time was associated with reduced physical activity, skeletal muscle weakness, and deconditioning (Albarrati et al. 2016). Zamboti et al. (2021) reported a moderate to strong correlation between the classic single-repetition TUG test and 6MWT distance in patients with ILD, whereas in our study, a strong correlation (*r* = −0.83) was found between the TUG-10 duration and 6MWT distance. Although both the classic TUG and the TUG-10 tests assess functional mobility, the TUG-10 involves multiple repetitions, adding muscle endurance to the other aspects of functional performance (i.e. muscle strength and mobility). Thus, the metabolic demand and the cumulative fatigue in TUG-10 are higher compared with the classic TUG, likely explaining the stronger correlation of the TUG-10 with the 6MWT observed in our study compared to the previous study.

### Study limitations

While this study provides valuable insights, some limitations should be acknowledged. The study sample was relatively small, and the majority of participants were diagnosed with fibrotic-ILD and specifically IPF, which limits the generalizability of the results to the broader spectrum of ILD. Future studies should aim to include a larger and more diverse sample of ILD patients, encompassing different disease subtypes. Another limitation is the use of pulse oximetry for assessing desaturation rather than arterial blood gas measurements. Although pulse oximetry is commonly used as a non-invasive surrogate for arterial blood oxygen saturation, it can overestimate true arterial oxygen saturation at rest and during exercise (Ascha et al. 2018; Donaldson et al. 2024). For this reason, we evaluated not only absolute SpO_2_nadir values, but also the magnitude of desaturation which may better reflect changes in oxygenation during exercise (Chuang et al. 2006).

### Clinical implications of TUG-10 and conclusions

The TUG-10 is a simple, time-efficient, and low-cost test that can detect exertional desaturation in patients with fibrotic-ILD. Despite its short duration, it elicits desaturation comparable to the 6MWT and CPET. The strong inverse association between TUG-10 and DL_CO_ suggests that the test can reflect pulmonary gas-exchange impairments. Given that the TUG-10 requires minimal equipment and space, and has a short duration, it can be used as a practical supplementary tool for assessment and monitoring exertional desaturation of patients with ILD in settings where time and space are limited. Further research is needed to explore its broader clinical applications, including its potential to monitor adaptations following pulmonary rehabilitation or other interventions or contribute to risk stratification in ILD management.

## Data Availability

The data that support the findings of this study are available from the corresponding author upon reasonable request.

## Abbreviations

CPET: Cardiopulmonary Exercise Test
DL_CO_: Diffusing Capacity of the Lungs for Carbon Monoxide
FVC: Forced Expiratory Volume
FEV1: Forced Expiratory Volume in 1 s
ILD: Interstitial Lung Diseases
IPF: Idiopathic Pulmonary Fibrosis
SpO_2_: Saturation from pulse oximetry
SpO_2_nadir: Lowest recorded oxygen saturation level
ΔSpO_2_: Difference in oxygen saturation
TUG-10: Time Up and Go-10 repetition
6MWT: 6-Minute Walk Test
VC: Vital Capacity
VO₂peak: Maximal oxygen uptake
